# Socioeconomic inequalities and family planning utilization among female adolescents in urban slums in Nigeria

**DOI:** 10.3389/fgwh.2022.838977

**Published:** 2022-08-02

**Authors:** Akanni Ibukun Akinyemi, Olutoyin Opeyemi Ikuteyijo, Jacob Wale Mobolaji, Temitope Erinfolami, Samuel O. Adebayo

**Affiliations:** ^1^Department of Demography and Social Statistics, Obafemi Awolowo University, Ile-Ife, Nigeria; ^2^Department of Epidemiology and Public Health, Swiss Tropical and Public Health Institute, University of Basel, Basel, Switzerland; ^3^Center for Research, Evaluation Resources and Development, Abuja, Nigeria

**Keywords:** socioeconomic inequalities, family planning, adolescent, urban slum, contraceptive, Nigeria

## Abstract

**Background/statement of problem:**

Family planning (FP) utilization is important for preventing unwanted pregnancy and achieving optimal reproductive health. However, the modern contraceptive prevalence rate (mCPR) among women of childbearing age is still low in many low- and middle-income countries (LMIC), particularly in Nigeria, despite interventions to increase access and utilization. The low mCPR has been associated with a high prevalence of unwanted pregnancy, unsafe abortion, sexually transmitted infections such as HIV/AIDS, and high maternal and infant mortality in LMIC. Despite existing studies associating high family planning utilization to urban settings relative to the rural areas, the socioeconomic inequality in urban settings, especially among adolescents in urban slums has been given less research attention. This study examines the role of socioeconomic inequality on family planning utilization among female adolescents of various ethnic backgrounds in urban slums in Nigeria.

**Methods:**

The study utilized data from the Adolescent Childbearing Survey (2019). A total sample of 2,035 female adolescents of ages 14–19 years who were not pregnant at the time of the study and were resident in selected slums. Associations between socioeconomic inequalities—measured by wealth index, social status, and education—and modern contraceptive use were examined using relative and slope inequality indices, and logistic regression models.

**Results:**

The results show that only 15% of the female adolescents in the North, and 19% in the South reported modern contraceptive use. While wealth index and education were important predictors of FP use among adolescents in southern urban slums, only education was important in the North. However, the relative and slope inequality indices further indicate that adolescents with no education and those in the lowest social status group use much fewer contraceptives compared to their counterparts with higher wealth and social statuses. Those with secondary/higher education and the highest social status group, respectively, were more disadvantaged in terms of FP utilization (Education: RII = 1.86, *p* < 0.05; 95% C.I. = 1.02–2.71; Social Status: RII = 1.97, *p* < 0.05; 95% C.I. = 1.26–2.68) with results showing a more marked level of disparity when disaggregated by North and South.

**Conclusion:**

The persistent socioeconomic inequalities among female adolescents in Nigeria, especially those in the urban slums, have continued to limit their utilization. Policy measure in education, communication and subsidized contraceptives should be intensified for vulnerable female adolescents in the slums.

## Background

Family planning utilization prevents adolescent pregnancy, improves health outcomes for women of childbearing age, and reduces maternal and infant deaths (1, 2). No <33 and 10% of the world's maternal and infant deaths, respectively, are preventable through contraceptive use (3). However, the modern contraceptive prevalence rate (mCPR) is still low in many low- and middle-income countries (LMICs) and has been associated with a high prevalence of unwanted pregnancy, unsafe abortion, sexually transmitted infections such as human immunodeficiency virus/acquired immunodeficiency syndrome (HIV/AIDS), and high maternal and infant mortality in countries. In 2015, about 15 million adolescent births were reported for LMICs (4). In a World Health Organization's (WHO) report (2020), globally, an estimated 21 million girls of ages 15–19 years, and 2 million under 15-year-old girls get pregnant, with about 16 million girls aged 15–19 years giving birth. About one-third of the adolescent pregnancies in many developing countries are unwanted and about one-third of these result in unsafe abortion (5, 6). Consequently, adolescents with unwanted pregnancies are more likely to drop out of school, experience child marriage, get stigmatized, rejected, or experience violence from partners, peers, and parents.

Despite the improved global awareness and contraceptive use, Sub-Saharan Africa (SSA) has the largest share of (high-risk) adolescent pregnancies and low mCPR (7, 8). Eliminating high-risk pregnancies can account for a 25% reduction in maternal mortality (9) and could in turn help in the reduction of the high fertility rate in the region. However, the low mCPR serves as a major barrier. A recent study investigating the unmet need for contraceptive use among adolescent girls and young women found a high prevalence of unmet needs for contraceptives among 10 high fertility countries in SSA (4). Despite the world's commitment to prioritize the needs of the vulnerable and underserved populations, through the Sustainable Development Goals (SDGs), about 13 million adolescent girls in developing countries still lack access to contraception (5). About half of these adolescents reside in Asia, and the Pacific and over 30% are in SSA. This evidence shows that the low contraceptive utilization in SSA is way behind achieving SDG 3, which focuses on ensuring good health for all, and SDG 5 on gender equality and empowerment of women and girls (10).

Nigeria is far behind in achieving the two SDGs (SDGs 3 and 4), which are of utmost importance to young people, despite being a signatory to the global commitment of leaving no one behind. The country's status is reflected in its low contraceptive statistics, which have implications for adolescent pregnancy. According to the latest report of Family Planning 2020 (FP2020), Nigeria has one of the lowest mCPR (11.7%) in SSA (11), with a high prevalence of unmet needs for contraceptives among adolescent girls (24.9%), and young women (18.7%) in the country (4). The government of Nigeria had made efforts to improve contraceptive uptake, yet no significant progress has been made. For instance, in 2018, the government developed a family planning blueprint with an overarching goal of increasing the country's mCPR from 15 to 36% by 2018, but the goal could not be achieved. A new blueprint was developed for 2020–2024 to increase the mCPR to 27%, but as of 2021, the mCPR remains low (11.7%).

Slum residents are exposed to poor healthcare services due to low socioeconomic and environmental conditions. A slum is characterized by substandard, congested, and overcrowded buildings and an unhealthy physical environment with poor or no social services (6, 12). A study on 23 SSA countries showed that maternal health care for the urban poor tends to be worse compared to those in rural areas (13). Though there has been an improvement over the years in terms of health care services, yet urban poor are the least to benefit.

Adolescents who reside in slum areas are vulnerable to adverse sexual and reproductive health (SRH) outcomes, due to low socioeconomic power to make a safe and informed decision (14, 15). These have social as well as SRH consequences including early sexual debut, unintended pregnancy, lack of continuing schooling, abortion, poverty, and violence (8, 16, 17). Adolescent pregnancies and births, for instance, are more pronounced among the poor and uneducated groups (13, 18). A study on the adolescents in the urban poor of Nairobi substantiated that the adolescents in the slum, especially those with limited parental influence, were vulnerable to risky sexual behaviors and adverse health outcomes (19). The vulnerabilities are also accentuated by the lack of or limited access to appropriate and quality SRH services in the slums. These findings signal the role of socioeconomic inequalities in family planning utilization among adolescents. Poverty is negatively associated with family planning utilization. This claim is reflected in the low level of contraceptive use in societies with low socioeconomic conditions compared to the high-income countries with high mCPR. With growing urbanization and the recent explosive expansion of slum areas, studies have shown a rise in unhealthy inequalities of health among slum dwellers, most especially, young people (14, 15).

Similarly, for the reason of accessibility and affordability, women with low socioeconomic status are less likely to use contraceptives compared to those with high status. According to a UNFPA report (5), lack of money and access to appropriate information to enable them to make informed choices are important barriers to contraceptive utilization among many adolescents. This finding is similar to a Malawian study which also associated low contraceptive utilization with poverty (20).

Moreover, education plays a vital role in women's health-seeking behavior. Educated women, who are likely to have access to media, and sexual and reproductive health education, are able to develop positive attitudes toward contraception and make informed decisions on their reproductive behaviors. Also, education helps women to secure gainful employment, earn a better income and increase the opportunity cost of leaving the gainful employment for childbearing (21). However, educated and high-income women are less likely to reside in the slums. It is, therefore, imperative to examine the role of educational inequality on contraceptive use among women, especially the adolescents who live in vulnerable communities.

Existing studies have examined factors associated with low mCPR which include poverty, low level of education, rural residence, socioeconomic status, and awareness of family planning methods (22, 23). Most of the studies have focused on all women, irrespective of their place of residence, with no consideration for adolescents, especially those in the slums, who are of varied socioeconomic class, largely unmarried, but engaging in high sexual activity and at risk of unwanted pregnancy. This study examines the role of socioeconomic disparities on contraceptive utilization of female adolescents in urban slums of Nigeria.

## Methods

### Study design

The study is cross-sectional, utilizing data from the Adolescents Childbearing Survey conducted in 2019 among female adolescents between the ages of 10–19 years who are sexually active; in a sexual relationship with the opposite sex, and not currently pregnant, living in urban slums. The survey focused on urban centers with a high population of adolescents in three Nigerian states namely Kaduna in the Northwest region, and Lagos and Oyo in the Southwest. These States were selected based on their relatively high records of mCPR (23) and high burdens of urban slums.

### Sampling design and sample size

Using the population counts from Nigeria's Geo-Referenced Infrastructure and Demographic Data for Development (GRID3) program (24), clusters of slum communities already identified from the literature were sampled using a multi-stage sampling design. The GRID3 population estimate shows the slum areas with a high population of adolescents and young people. The slum areas that were in urban centers were selected as the study clusters. Households with female adolescents were randomly selected until the cluster sample size was attained. A female adolescent was randomly selected in households with more than one eligible female respondent to ensure community representativeness. The study utilized a total sample of 2,035 childbearing female adolescents from the three states (Kaduna = 591, Lagos = 708, and Oyo = 736), who were sexually active but non-pregnant, married, or unmarried.

### Variable measurement

The outcome variable for the study was the use of modern contraceptives, which was determined based on a respondent's report of current use of any modern contraceptive method. The respondents were asked about their current contraceptive method with a “yes” or “no” response for each modern contraceptive method listed including male or female condom, injection, pill, implant, or Intra-Uterine Device (IUD).

The main independent variable for this study is the socioeconomic inequalities of the respondents. The variable was measured using the household wealth index, social status, and adolescents' highest level of educational attainment. We adapted the Demographic and Health Survey (DHS) approach to computing the household wealth index (25) by using an aggregate measure of 32 household items possessed in the respondent's household. These include the availability of electricity, possession by any household member of a wall clock, a radio, color television, a mobile phone, a refrigerator, a freezer, an electric generator and/or an inverter, a washing machine, a computer/tablet computer, a photo camera, a video Deck/DVD/VCD, a cable network like DSTV, a sewing machine, a bed with foam, a dining table, a cabinet/cupboard, access to the internet on any device, a wristwatch, a bicycle, a motorcycle or motor scooter, an animal-drawn cart, a car or truck, a boat with a motor, a boat without a motor, a plot of Land. It also included variables on the availability of improved wall, roof, and floor materials, as well as improved drinking water, toilet facilities, and fuel for cooking. We computed the inter-item correlations or covariance for all pairs of variables considered, established internal consistency using the Cronbach's alpha statistic, and eventually used principal component analysis (PCA) to transform the final score of these variables into a quintile (poorest, poorer, middle, richer, and richest) to reflect household wealth. Social status on the other hand was measured using the aggregate of four variables that are meant to predispose respondents to social protection opportunities and aid their social participation in their immediate environment. They included (i) ownership of a bank account (financial); (ii) ownership of any of a national identification card, a voter's card, a driver's license, or an international passport (identification); (iii) ownership of a health or hospital registration card; and (iv) ownership of a mobile phone. All these items were coded as “1” if a respondent had them and “0” otherwise. The eventual aggregate score was categorized into a tertile: low, middle, and high. Other variables including age, marital status, religion, employment status, and age at first sex were used as covariates.

### Data analysis

The data was analyzed at three levels (univariate, bivariate, and multivariable). The univariate analysis involved frequency and percentage distribution used to report the prevalence of modern contraceptive utilization among the respondents. At the bivariate level, we examined the association between the equity variables and the use of modern contraceptives using the Chi-Squared test of independence. We also used equiplot to identify and visualize inequalities in the uptake of modern contraceptives across the ordered categories of wealth quintile, social status, and education. The equiplot is an analytical tool that allows for the visualization of the coverage of an outcome of interest across one or more equity indicators of interest (26). All our analyses are stratified by study sites (slums) in the North and South. Controlling for other background variables, we first used a logistic regression model to assess the odds of using modern contraceptives in the lowest category of each equity variable compared with the higher category. All analyses were based on a 95% confidence interval, excluding missing data. Further, examining inequalities in the slums, two measures of inequality were estimated—the relative index of inequality (RII) and the slope index of inequality (SII). While the RII reflects relative inequalities, the SII measures absolute inequalities. The RII was used to account for the relative difference in the sub-categories of the three equity variables. It is a measure that is based on a weighted linear regression that relates to the use of modern contraceptives with the relative position of individuals on the distribution of an equity variable. This can be interpreted as the prevalence rate of using a modern contraceptive method at the bottom categories of the equity variables compared with the prevalence rate at the top of the hierarchy of the same variables. The SII on the other hand is a predictor of absolute inequalities, which measures the difference between the predicted value of an outcome in both endpoints of the distribution of an explanatory (equity) variable. It is the absolute equivalent to the RII and is computed as the slope of the weighted linear regression. It represents the average difference in the use of modern contraception between those at the top of the hierarchy compared with those at the bottom for each of the equity variables. It varies between −1 and +1, where 0 represents the absence of inequalities. A positive SII value indicates that the use of modern contraceptives is concentrated in the better half [i.e., the richest group(s) if we analyze inequalities according to wealth], and thus, reflecting unequal access for those in the poorer half. More details on the SII and RII are published elsewhere (27–29). All analyses were done at a 95% confidence level using Stata 15.1.

## Results

Table 1: overall, 77.9% of the study respondents had secondary education. This is however slightly higher in the South than in the North. Regarding their household wealth, 24.5 and 21.1% of the study participants in the North were from the poorest and richest wealth quintiles, respectively, compared with their southern counterparts, 20.6 and 16.2%, respectively. About three out of every five (60%) of the study respondents were of low social status, compared with about 14% in the highest social status with North-South variations. The respondents aged 18 years accounted for the largest proportion of women (38.9%) sampled, and about 35.2% were currently employed. There was a preponderance of Muslims in the sample, accounting for slightly more than half (55%) in the South and about 71% in the North. Similarly, the majority (69%) of the respondents are married in the two study divides. Slightly over one-fifth of the respondents had debuted sex before age 15 in both regions, however, only 18% of the women from the southern region and 16% in the North use modern contraceptives.

**Table 1:** Distribution of slum-dwelling adolescents by equity variables and the background characteristics of adolescent girls in selected urban slums in Nigeria.

	**South**	**North**	**Total**
	***N* = 1,444 (%)**	***N* = 591 (%)**	***N* = 2,035 (%)**
Education			
None	5.4	6.8	5.8
Primary	11.1	13.2	11.7
Secondary	79.3	74.6	77.9
Higher	4.2	5.4	4.6
Household quintile			
Poorest (Q1)	20.6	24.5	21.7
Poorer (Q2)	20.8	20.0	20.6
Middle (Q3)	25.8	21.3	24.5
Richer (Q4)	16.6	13.0	15.5
Richest (Q5)	16.2	21.2	17.6
Social status			
Low	63.0	52.3	59.9
Middle	24.0	32.7	26.5
High	13.0	15.1	13.6
Age			
14	1.9	2.9	2.2
15	3.3	5.6	3.9
16	6.9	8.3	7.3
17	12.6	13.9	13.0
18	38.2	40.6	38.9
19	37.1	28.8	34.7
Employed			
No	61.3	73.3	64.8
Yes	38.7	26.7	35.2
Muslim			
No	44.8	29.3	40.3
Yes	55.2	70.7	59.7
Married			
No	30.8	31.0	30.9
Yes	69.2	69.0	69.1
First sex before age 15			
No	78.8	78.0	78.6
Yes	21.2	22.0	21.4
Using modern contraceptives			
No	82.1	84.1	82.7
Yes	17.9	15.9	17.3

Table 2 shows the bivariate association between women's background characteristics and modern contraceptive use in the study area. Unlike in the South where education had no significant association with the use of FP, results from the North shows a significant association between the respondent's level of education and modern contraceptive use (*p* = 0.025), which increased with higher levels of education. Across both regions, however, the association between women's household wealth and modern contraceptive use was not statistically significant. Conversely, modern contraceptive utilization increased with higher social status in the overall data. The association is statistically significant only in the South (*p* = 0.001).

**Table 2:** Bivariate association between modern contraceptive use, equity variables, and background characteristics of adolescent girls in selected urban slums in Nigeria.

	**South**		**North**		**Total**	
	***N***	***n* (%)**	***N***	***n* (%)**	***N***	***n* (%)**
Overall	1,444	259 (17.9%)	591	94 (15.9%)	2,035	353 (17.3%)
**Equity variables**						
Education						
None	78	8 (10.3%)	40	2 (5%)	118	10 (8.5%)
Primary	160	27 (16.9%)	78	8 (10.3%)	238	35 (14.7%)
Secondary	1,145	215 (18.8%)	441	75 (17%)	1,586	290 (18.3%)
Higher	61	9 (14.8%)	32	9 (28.1%)	93	18 (19.4%)
*Chi*^2^ (*p*-value)		*4.218 (0.239)*		*9.390 (0.025)*		*8.871 (0.031)*
Wealth quintile						
Poorest (Q1)	297	62 (20.9%)	145	25 (17.2%)	442	87 (19.7%)
Poorer (Q2)	301	40 (13.3%)	118	22 (18.6%)	419	62 (14.8%)
Middle (Q3)	373	61 (16.4%)	126	18 (14.3%)	499	79 (15.8%)
Richer (Q4)	239	52 (21.8%)	77	11 (14.3%)	316	63 (19.9%)
Richest (Q5)	234	44 (18.8%)	125	18 (14.4%)	359	62 (17.3%)
*Chi*^2^ (*p*-value)		*9.284 (0.054)*		*1.465 (0.83)*		*5.862 (0.210)*
Social status						
Low	909	136 (15.0%)	309	43 (13.9%)	1,218	179 (14.7%)
Middle	347	83 (23.9%)	193	34 (17.6%)	540	117 (21.7%)
High	188	40 (21.3%)	89	17 (19.1%)	277	57 (20.6%)
*Chi*^2^ (*p*-value)		*15.329 (0.000)*		*2.016 (0.365)*		*15.014 (0.001)*
**Background characteristics**						
Age						
14	28	3 (10.7%)	17	1 (5.9%)	45	4 (8.9%)
15	47	7 (14.9%)	33	7 (21.2%)	80	14 (17.5%)
16	99	20 (20.2%)	49	4 (8.2%)	148	24 (16.2%)
17	182	34 (18.7%)	82	10 (12.2%)	264	44 (16.7%)
18	552	107 (19.4%)	240	47 (19.6%)	792	154 (19.4%)
19	536	88 (16.4%)	170	25 (14.7%)	706	113 (16%)
*Chi*^2^ (*p*-value)		*3.327 (0.650)*		*7.622 (0.178)*		*5.780 (0.328)*
Employed						
No	885	143 (16.2%)	433	58 (13.4%)	1,318	201 (15.3%)
Yes	559	116 (20.8%)	158	36 (22.8%)	717	152 (21.2%)
*Chi*^2^ (*p*-value)		*4.910 (0.027)*		*7.631 (0.006)*		*11.463 (0.001)*
Muslim						
No	647	105 (16.2%)	173	52 (30.1%)	820	157 (19.1%)
Yes	797	154 (19.3%)	418	42 (10%)	1,215	196 (16.1%)
*Chi*^2^ (*p*-value)		*2.322 (0.128)*		*36.628 (0.000)*		*3.103 (0.078)*
Married						
No	445	62 (13.9%)	183	30 (16.4%)	628	92 (14.6%)
Yes	999	197 (19.7%)	408	64 (15.7%)	1,407	261 (18.6%)
*Chi*^2^ (*p*-value)		*7.005 (0.008)*		*0.047 (0.828)*		*4.607 (0.032)*
First sex before age 15						
No	1,138	199 (17.5%)	461	69 (15%)	1,599	268 (16.8%)
Yes	306	60 (19.6%)	130	25 (19.2%)	436	85 (19.5%)
*Chi*^2^ (*p*-value)		*0.737 (0.391)*		*1.378 (0.240)*		*1.787 (0.181)*

While the results further show that the age of respondents and their age at first sex (sexual debut before age 15) were not associated with contraceptive use in both regions, other covariates such as employment status, religion, and marital status had mixed results. For instance, though being employed shows significant association with modern contraceptive use in both regions, religion shows significant association only in the North compared with marital status only in the South.

**Figure 1:**
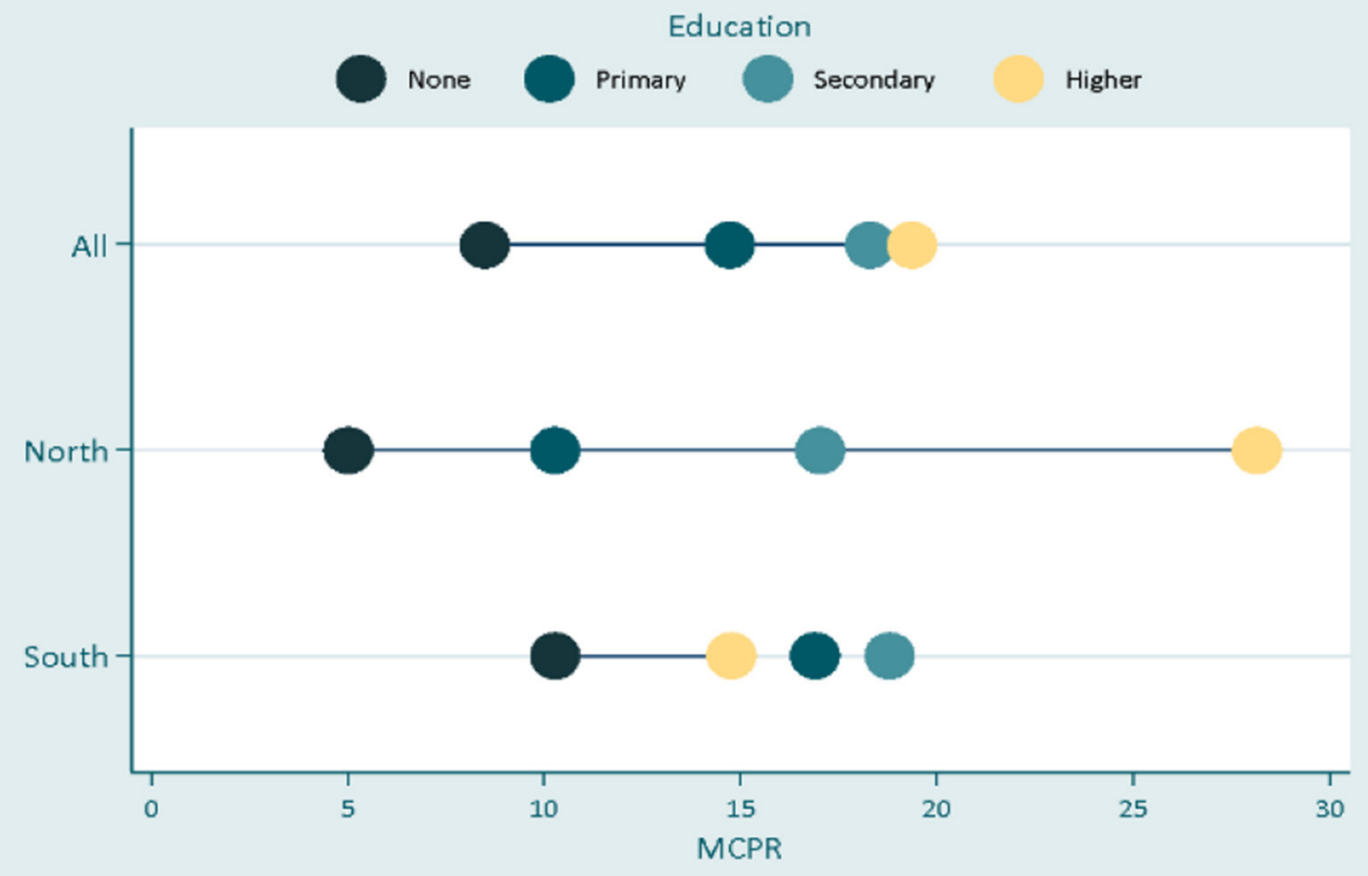
Inequalities in contraceptives uptake by education.

In Figure 1, the result shows that there was a significant disparity in modern contraceptive uptake by educational status, across the two divides. In the South, inequality in contraceptive uptake was random across levels of education with about 5% more female adolescents with tertiary education using FP compared with their completely uneducated counterparts, while adolescents with primary and secondary education used modern contraceptives more than those with tertiary education. This reflects a low level of inequality between the least educated and the most educated groups. In the North, however, education made so much difference in the uptake of modern contraceptive methods as the rate of modern contraceptive use was significantly higher for respondents in every successive higher educational category. In addition, there exists a wide gap between the uneducated women and the tertiary-educated women indicating a high level of inequality for women with lower education in the North.

**Figure 2:**
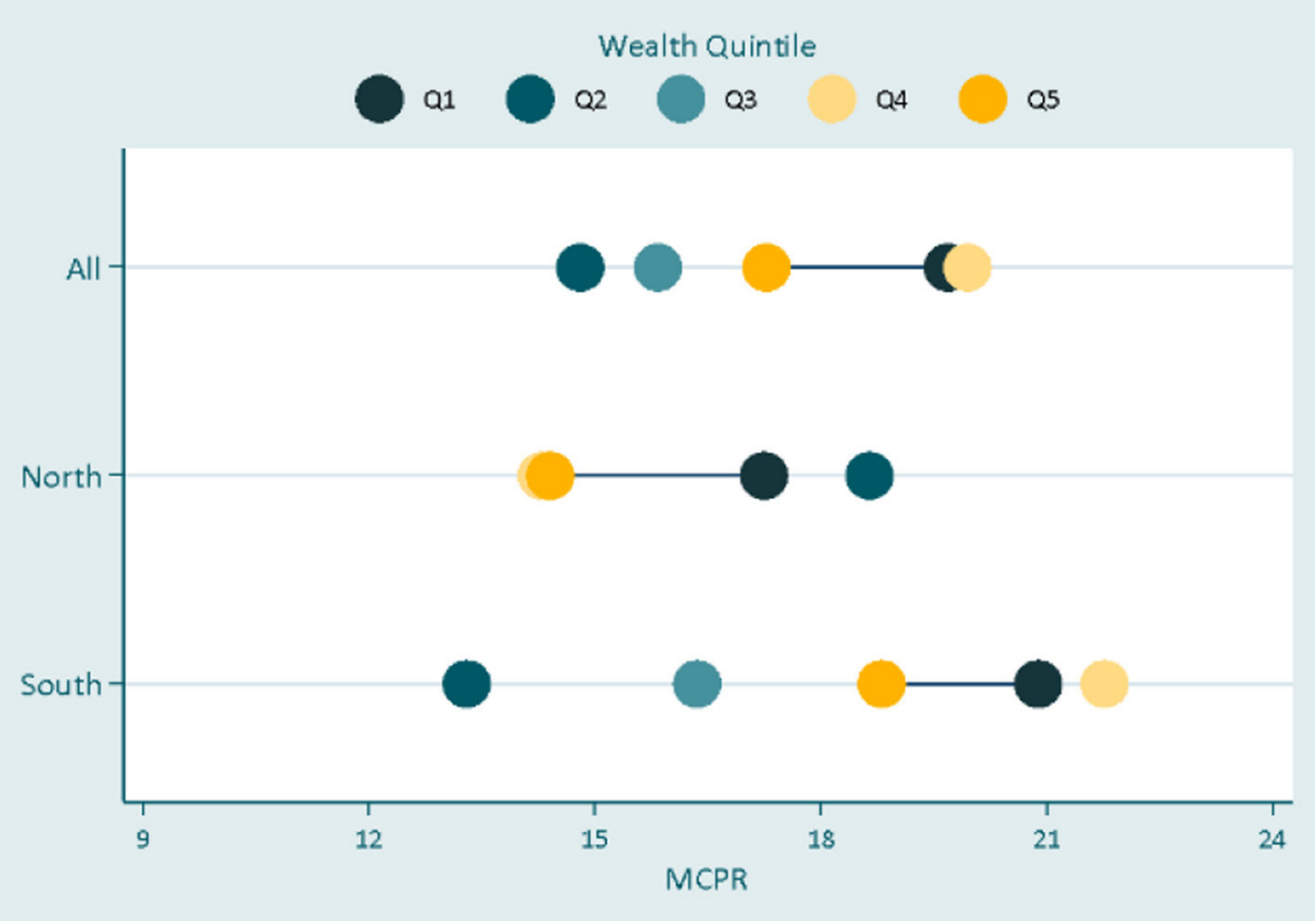
Inequalities in contraceptives uptake by household wealth.

**Figure 3:**
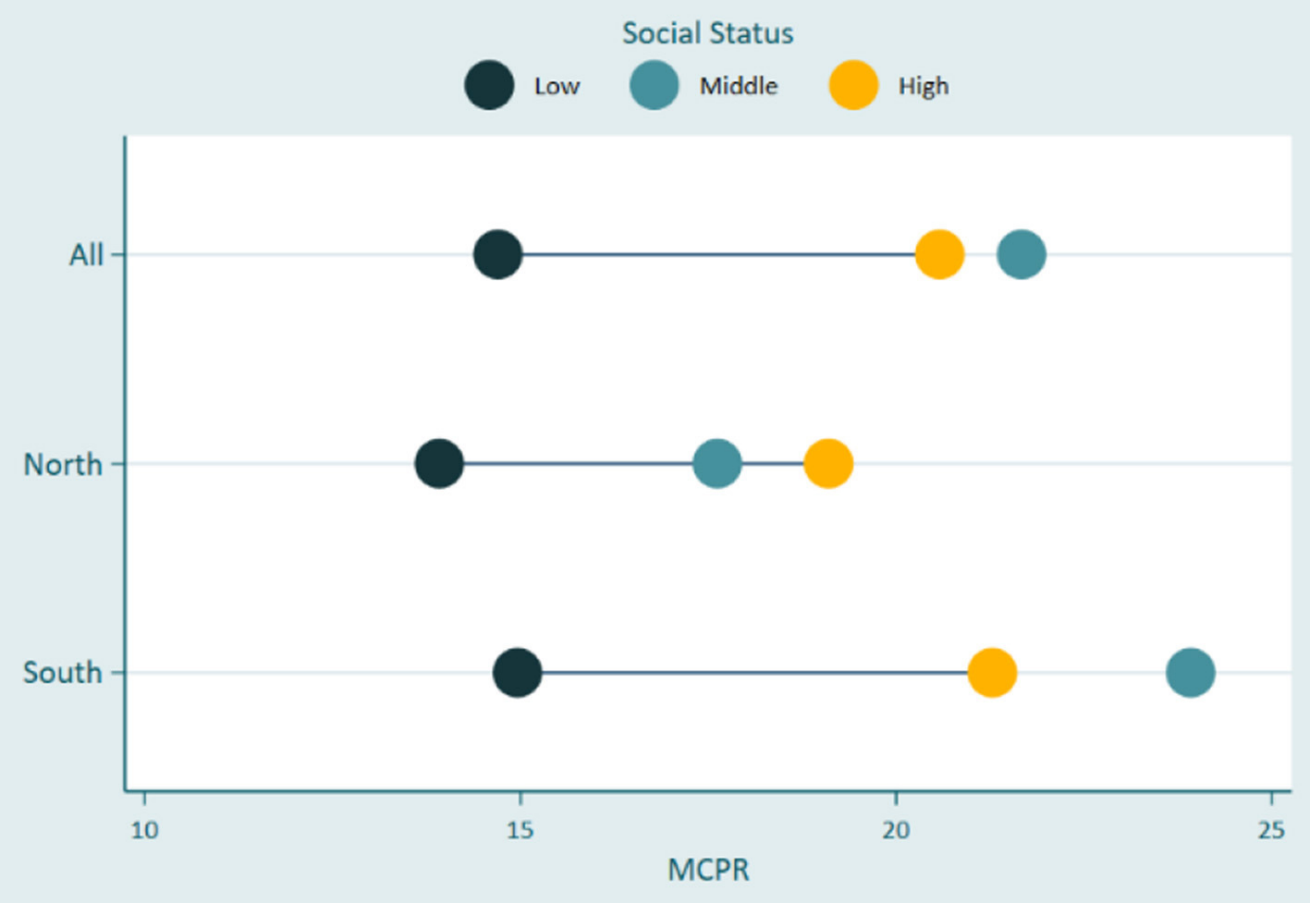
Inequalities in contraceptives uptake by social status.

In Figure 2, the result shows that there was no noticeable difference in modern contraceptive uptake across the wealth quintiles as a larger proportion of adolescents in the poorest groups consistently used modern contraceptives compared with their counterparts in the richest wealth groups. In Figure 3, there was just about the same rate of contraceptive use in the overall data irrespective of the inequality in social status. However, while the respondents with low social status demonstrated the least contraceptive use in the North and South, respondents in the middle group had used more contraceptives in the south where they demonstrated even more uptake than respondents in the highest social status. The results in Table 3 show the adjusted odds ratios (AOR) and 95% confidence intervals (95% CI) estimated using multivariable logistic regression models to quantify associations between equity variables and modern contraceptive use. Across the equity indicators, while education did not predict modern contraceptive use in the South, the result shows that the odds of using modern contraception among women with tertiary education in the Northern region of the study area was about six-fold higher (OR = 6.27; *p* < 0.05; 95% C.I. = 1.08–36.28) compared to the reference category- adolescents with no formal education. In the overall data, women from poorer, middle, and richest households were significantly less likely to use modern contraceptives (poorer: OR = 0.63, *p* < 0.05, 95% C.I. = 0.44–0.91; Middle: OR = 0.64, *p* < 0.05; 95% C.I. = 0.45–0.91; richest: OR = 0.59, *p* < 0.05; 95% C.I. = 0.40–0.89) compared to their counterparts from the poorest households. These associations were similar among women in the poorer and middle wealth quintile in the South. Lastly, women with middle and high social statuses were 54 and 50%, respectively, more likely to use modern contraceptives than their peers with low social status in the overall data. This association was consistent in the South (Middle: OR = 1.77, *p* < 0.01, 95% C.I. = 1.27–2.47; High: OR = 1.60, *p* < 0.05, 95% C.I. = 1.02–2.50), but insignificant in the North. All the covariates including age, employment, marital status, and sexual debut before 15 were significantly associated with increased contraceptive use, except in the South. However, being a Muslim in the North was associated with about 90% lower odds of using a modern contraceptive.

**Table 3:** Multivariable logistic regression models of associations between background characteristics, equity variables, and modern contraceptive use of adolescent girls in selected urban slums in Nigeria.

**Variables**	**South**		**North**		**Total**	
	**AOR**	**95% CI**	**AOR**	**95% CI**	**AOR**	**95% CI**
**Equity variables**						
Education						
None	*Ref*.	–	*Ref*.	–	*Ref*.	–
Primary	1.85	[0.78–4.35]	1.71	[0.33–8.85]	2.07	[0.98–4.40]
Secondary	2.14	[0.98–4.66]	1.8	[0.39–8.22]	2.81**	[1.42–5.54]
Tertiary	1.37	[0.47–3.99]	6.27*	[1.08–36.28]	2.68*	[1.12–6.41]
Wealth quintile						
Q1	*Ref*.	–	*Ref*.	–	*Ref*.	–
Q2	0.48**	[0.31–0.76]	1.31	[0.65–2.66]	0.63*	[0.44–0.91]
Q3	0.61*	[0.41–0.93]	0.9	[0.42–1.92]	0.64*	[0.45–0.91]
Q4	0.76	[0.48–1.19]	0.85	[0.34–2.10]	0.74	[0.50–1.10]
Q5	0.63	[0.39–1.02]	0.56	[0.25–1.25]	0.59*	[0.40–0.89]
Social status						
Low	*Ref*.	–	*Ref*.	–	*Ref*.	–
Middle	1.77***	[1.27–2.47]	1.8	[0.99–3.26]	1.54**	[1.17–2.03]
High	1.60*	[1.02–2.50]	2.17	[0.96–4.89]	1.50*	[1.03–2.18]
**Background characteristics**						
Age						
14	*Ref*.	–	*Ref*.	–	*Ref*.	–
15	1.57	[0.36–6.82]	4.38	[0.45–42.75]	2.56	[0.77–8.43]
16	2.34	[0.63–8.79]	1.08	[0.10–11.92]	2.3	[0.74–7.15]
17	2.16	[0.59–7.88]	3.61	[0.39–33.25]	2.61	[0.86–7.87]
18	2.32	[0.65–8.32]	6.46	[0.74–56.77]	3.28*	[1.10–9.72]
19	1.98	[0.54–7.23]	5.13	[0.55–47.68]	2.77	[0.92–8.37]
Employed	1.2	[0.91–1.60]	2.05**	[1.22–3.45]	1.37**	[1.08–1.75]
Muslim	1.2	[0.90–1.60]	0.10***	[0.05–0.19]	0.75*	[0.59–0.97]
Married	1.39*	[1.01–1.91]	2.84**	[1.47–5.49]	1.37*	[1.04–1.81]
Had first sex before 15	1.19	[0.81–1.76]	2.38*	[1.18–4.78]	1.40*	[1.00–1.95]
Constant	0.04***	[0.01–0.17]	0.02**	[0.00–0.34]	0.03***	[0.01–0.09]
Observations	1,444		591		2,035	

*** *p < 0.001*,

** *p < 0.01*,

* *p < 0.05; AOR, adjusted odds ratios; CI, confidence intervals*.

The results from the analysis of relative and slope indices of inequality, as illustrated in Table 4, further shows the disparity in the uptake of modern contraceptives by socioeconomic indicators in the study areas. The results show the education-, wealth-, and social-related RII and SII in modern contraceptive utilization. According to the results in model 1, the SII result indicates that the difference in the mCPR of the female adolescents with no formal education and their counterparts with education was 22% in the North, while that of low and highest social statuses was 14%. In model 2, the mCPR among the respondents with no formal education was 1.35 times the mCPR among adolescents with the highest education in the south (β = 1.35, *p* < 0.05, 95% C.I. = 0.65–2.05), whereas the disparity was by 4.13 higher in the North (β = 4.13, *p* < 0.05, 95% C.I. = 0.34–7.92). The mCPR result is similar for social status in both regions. However, the mCPR among the female adolescents in the poorest wealth quintile was 1.11 times higher (β = 1.11, *p* < 0.05, 95% C.I. = 0.66–1.56) than their counterparts in the richest wealth quintile.

**Table 4:** Measures of inequality in the uptake of modern contraceptives among adolescent girls in selected urban slums in Nigeria.

**Equity variables**		**Model 1** **SII**		**Model 2 RII**	
		**β**	**95% CI**	**β**	**95% CI**
Educational status	South	0.05	[−0.04–0.15]	1.35***	[0.65–2.05]
	North	0.22***	[0.08–0.37]	4.13*	[0.34–7.92]
	Total	0.11***	[0.03–0.19]	1.86***	[1.02–2.71]
Household wealth	South	0.02	[−0.05–0.09]	1.11***	[0.66–1.56]
	North	−0.05	[−0.15–0.05]	0.73***	[0.25–1.21]
	Total	0.00	[−0.06–0.06]	0.98***	[0.64–1.32]
Social status	South	0.14***	[0.06–0.21]	2.19***	[1.26–3.11]
	North	0.08	[−0.03–0.19]	1.66***	[0.50–2.82]
	Total	0.12***	[0.05–0.18]	1.97***	[1.26–2.68]

*** *p < 0.001*,

** *p < 0.01*,

* *p < 0.05*.

## Discussion

Globally, socioeconomic indicators have played important roles in health behaviors and outcomes. This study examines the roles of socioeconomic inequalities in the uptake of modern contraceptives among adolescents in selected urban slums of northern and southern Nigeria. Generally, the utilization of modern contraceptive methods in the selected urban slums was much higher than was reported among young people of the same age group from the Demographic and Health Survey (DHS) (30) that was conducted about the same time (this study: 17.9% in the south vs. 15.9% in the north; DHS: 1.4% in the South vs. 2.3% in the North). On the one hand, the disparities in the findings of our study and the DHS report could be linked to the high sexual activities among adolescents who are slum dwellers compared to their counterparts who are non-slum dwellers with no need of contraceptive use (31). On the other hand, one might argue that slum-dwellers are using contraceptives more in a bid to deal with the deterioration of their environment (32). Though the DHS is a state-wide survey while this study is only specific to selected slum areas within each state, our study, nevertheless, highlights how the slum-dwelling adolescents compare with the general population of their peers, many of whom are non-slum dwellers. The study also shows the disparities in equity in terms of household wealth, individual woman's education, and social status.

An important finding was that, unlike in the Southern slums, household wealth in the Northern slums was not important in the use of contraceptives among the female adolescents, as the use of modern contraceptive methods was random across wealth groups in the north. In the south, however, higher wealth status predicts lower use of contraceptives. This finding may be due to a likelihood of low levels of empowerment among young girls in Northern Nigeria, in which case, they are married to older partners and they do not usually possess the agency to participate, either solely or partially, in the decision to utilize modern contraceptives, irrespective of the relative level of wealth in their household (33). This might also be an indication of the little relevance of relative household wealth on reproductive health in communities where the rich among the poor are also poor because of the collective orientation they share. Though this specific finding contrasts with a glut of earlier studies in Nigeria and elsewhere that have established the increased use of modern contraceptives among the wealthier and least deprived groups (33–36), it, however, compares with a few other studies that have found higher contraceptive prevalence among women residing in resource-poor communities (37, 38) indicating the importance of zeroing in on specific vulnerable groups to understand their specific needs.

Similar to the findings on wealth, another important finding was the significantly positive effect of higher social statuses on the use of modern contraception in the slums. For instance, in Southern Nigeria, our study shows an increased level of contraceptive use among the respondents who possessed the catalysts of access to social protection opportunities and social participation within their communities. This subtly highlights the importance of social participation in health decision-making and is in line with findings from studies that have demonstrated higher subjective health among people who engaged in social and civic participation compared with those who do not (39, 40). Of more relevance is what this finding means for slum-dwelling adolescents. Given the levels of deprivation inherent on them by virtue of their environment, efforts should be targeted at this demographic to ensure equitable distribution of common amenities to help slum dwellers continue to make healthy decisions and lead healthy lives.

Furthermore, our study shows the positive effect of education on contraceptive use among slum-dwelling adolescents. Higher levels of education significantly increased the utilization of modern contraceptives, although this influence was more significant in the North. This indicates inequalities in contraceptive use among the uneducated adolescents in the slums generally, and, more specifically, in the Northern urban slums. These findings compares with earlier studies (41, 42). This study highlights the key findings of major facilitators of inequalities that exist among the adolescent in the slums—education, wealth quantile, and social status, and its findings are therefore imperative for understanding the influence of multiple burdens of vulnerability—disadvantaged socioeconomic condition of dependent persons residing in vulnerable communities—on contraceptive utilization among young women.

### Strength and limitation

The study sample was limited to only adolescents who were not currently pregnant at the time of the survey. Their experiences were not captured. The measurement of social status in this study is relative to the available data component, which focuses more on household wealth.

### Conclusion

Social and economic inequalities in the urban space are major determinants of contraceptive utilization. Inequalities of educational levels exist among adolescents in the slums remain a major factor, alongside wealth quantile and other social statuses which contributed to the level of utilization of contraceptives. Nonetheless, improving access to education for female adolescents will therefore increase the uptake of contraceptives among this category of young people. Sexual education and adolescent youth-friendly sexual and reproductive health services (AYF-SRHS) will contribute as drivers to increasing the utilization of the services.

### Implication for program policy and research

Efforts to improve the access of adolescents to contraceptives have recently taken the front burner in the Nigerian health sector. However, the persistence of socio-economic inequalities among this group, most especially, in the urban slums has continued to limit their utilization. As a policy measure, education and communication programs should be targeted toward vulnerable female adolescents in the slum settlements in Nigeria.

## Data Availability Statement

In order to protect the privacy of the research participants, the supporting data for the findings of this study are available only on request from the corresponding author.

## Ethics Statement

The studies involving human participants were reviewed and approved by the National Health Research Ethics Committee of Nigeria (NHREC), which approved this study and the protocol on May 25, 2018, with IRB number NHREC/01/01/2007. A letter of approval for the publication was granted by Harvard T.H. Chan School of Public Health, IRB18-1385 August 27, 2018. Written informed consent to participate in this study was provided by the participants' legal guardian/next of kin.

## Author contributions

AA conceptualized and reviewed and revised the manuscript. OI, JM, and SA wrote the background. SA wrote the methodology. TE analyzed the data and wrote the result with JM. All authors contributed to the discussion and reviewed the final manuscript.

## Funding

JM is a fellow of the Consortium for Advanced Research Training in Africa (CARTA). CARTA is jointly led by the African Population and Health Research Center and the University of the Witwatersrand and funded by the Carnegie Corporation of New York (Grant No. G-19-57145), Sida (Grant No:54100113), Uppsala Monitoring Center, Norwegian Agency for Development Cooperation (Norad), and by the Wellcome Trust (reference no. 107768/Z/15/Z) and the UK Foreign, Commonwealth and Development Office, with support from the Developing Excellence in Leadership, Training and Science in Africa (DELTAS Africa) programme. OI is a recipient of European Union's Horizon 2020 research and innovation program under the Marie Sklodowska-Curie grant agreement No. 801076, through the SSPH + Global and BOTNAR Foundation, and Ph.D. Fellowship in Public Health Sciences (GlobalP3HS) of the Swiss School of Public Health.

## Conflict of interest

The authors declare that the research was conducted in the absence of any commercial or financial relationships that could be construed as a potential conflict of interest.

## Publisher's note

All claims expressed in this article are solely those of the authors and do not necessarily represent those of their affiliated organizations, or those of the publisher, the editors and the reviewers. Any product that may be evaluated in this article, or claim that may be made by its manufacturer, is not guaranteed or endorsed by the publisher.

## Author disclaimer

The statements made and views expressed are solely the responsibility of the Fellow. For the purpose of open access, the author has applied a CC BY public copyright license to any author accepted manuscript version arising from this submission.

## Figures and Tables

**Figure:**
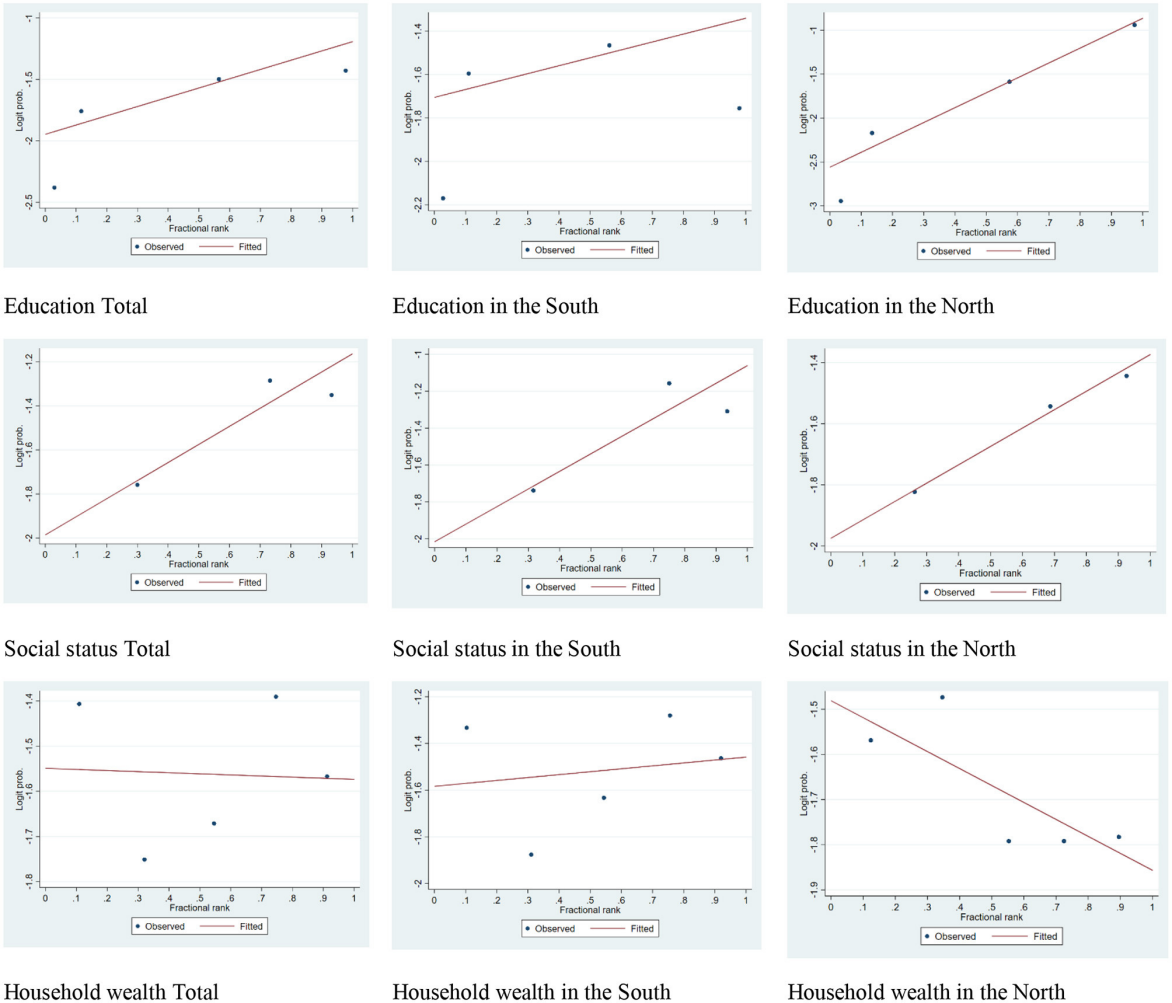


## Appendix

In the SII plot, the dots represent the values of the outcome variable in each group of the ranking variable. It is interpreted based on the direction of the outcome according to the ranking variable. For instance, when the outcome increases according to the ranking variable, providing a positive SII estimate, there is a higher inequality among the less advantaged groups.
